# The DCM Project Portal: A direct-to-participant platform of The DCM Research Project^☆^

**DOI:** 10.1016/j.ahjo.2023.100356

**Published:** 2023-12-27

**Authors:** Elizabeth S. Jordan, Phoenix L. Grover, Jay Lin, Carl A. Starkey, Elizabeth A. Finley, Hanyu Ni, Ray E. Hershberger

**Affiliations:** a The Davis Heart and Lung Research Institute, The Ohio State University, Columbus, OH, United States of America; b Division of Human Genetics, Department of Internal Medicine, The Ohio State University, Columbus, OH, United States of America; c Division of Cardiovascular Medicine, Department of Internal Medicine, The Ohio State University, Columbus, OH, United States of America

**Keywords:** Research design, Family members, Dilated cardiomyopathy

## Abstract

**Study objective:**

To develop a digital platform to conduct family-based, dilated cardiomyopathy (DCM) genetic research.

**Design:**

The DCM Project Portal, a direct-to-participant electronic recruitment, consent, and communication tool, was designed using prior experience with traditional enrollment methods and characteristics and feedback of current participants.

**Participants:**

DCM patients (probands) and their family members enrolled from June 7, 2016 to March 15, 2020 at 25 US advanced heart failure programs.

**Results:**

The portal was designed as a self-guided, three module (registration, eligibility, and consent) process with supporting informational and messaging resources integrated throughout. The experience is tailored to user type and the format adaptable with programmatic growth. Characteristics of participants of the recently completed DCM Precision Medicine Study were assessed as an exemplary user population. A majority of the diverse (34 % non-Hispanic Black (NHE-B), 9.1 % Hispanic; 53.6 % female) proband (*n* = 1223) and family member (*n* = 1781) participants aged ≥18 years reported *not at all* or *rarely* having problems learning about their health from written information (81 %) and a high confidence in completing medical forms (77.2 % *very much* or *often* confident), supporting a self-guided model. A majority of participants across age and race-ethnicity groups reported internet access, with highest rates of no reported access in those ≥77 years (31.9 %), NHE-B (25.2 %), and Hispanic (22.9 %), a similar pattern to those reported by the US Census Bureau as of 2021.

**Conclusions:**

The portal is an example of a digital approach to family-based genetic research that offers opportunity to improve access and efficiency of research operations.

## 1 Introduction

Clinical genetics research can be conducted in case-based designs, where the unit of investigation is a patient with a phenotype of interest. This model has been fruitful for gene discovery and genotype-phenotype correlations, and particularly for high penetrance genes and variants. However, in complex cardiovascular genetic disease, case-only approaches may be insufficient. Shifting the paradigm to viewing a family as the unit of investigation, i.e., the patient with the phenotype of interest *and* their relatives, has promise to provide a more comprehensive view of shared rare and common variant, epigenic, environmental, and other factors within a family that contribute to the marked clinical and genetic complexity in heritable cardiovascular disease [1]. Although a family-based approach is recommended in the clinical genetic evaluation of cardiomyopathy [2], recruiting a family unit is labor-intensive and challenging, and persists as a major barrier to implementation of a family-based research design.

The Dilated Cardiomyopathy (DCM) Research Project is a family-based research program established in the 1990s that aims to investigate the genetics of DCM, a condition characterized by left ventricular enlargement and systolic dysfunction and a leading cause of heart failure [1,3]. When the cause of DCM remains unknown after a thorough clinical evaluation, an underlying genetic cause is suspected, but clinical genetic testing identifies a cause in only about one-third of cases [4,5]. The unsolved clinical and genetic complexity of DCM in patients and their family members drives DCM Research Project investigations.

The DCM Precision Medicine study, a recently completed multi-site, National Institutes of Health-funded study conducted by the DCM Research Project, enrolled >1200 DCM patients (probands) and >2000 of their family members from diverse racial and ethnic backgrounds to perform clinical DCM screening and genetic testing [[6], [7], [8]]. The aims of the DCM Precision Medicine study were to test the hypothesis that DCM has substantial genetic basis and to evaluate the effectiveness of a family communication intervention in improving the uptake of family member clinical screening [7,8]. Data from family units has been critical to the emerging findings from the Precision Medicine study [6,9,10], but despite these initial informative reports and others from this study [11,12], many questions remain. To continue to decipher the complexity of DCM genetics, an essential step toward advancing preventive and therapeutic approaches to genetic DCM, participation of a much larger number of families from diverse backgrounds is needed [[13], [14], [15]]. The DCM Research Project aims to achieve this through the aggregation of a much larger cohort of DCM families, nominally 10,000, for the DCM Discovery Study.

**Fig. 1:**
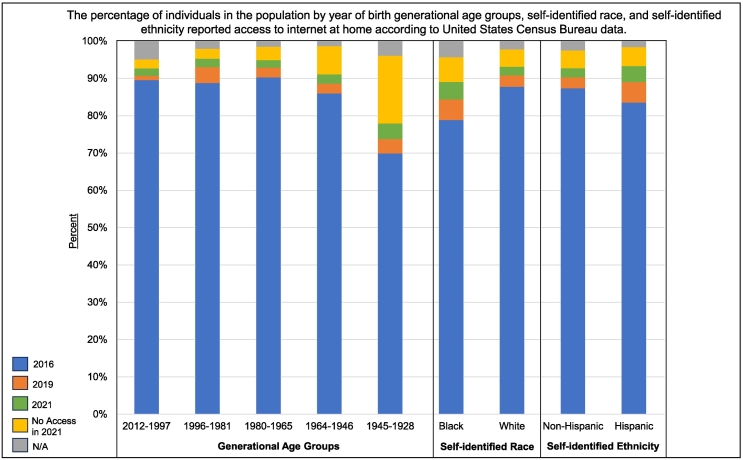
Reported internet access in the US population in 2016, 2019, and 2021 according to US Census data. The percentage of individuals in the population within generational age groups by year of birth, self-identified race, and self-identified ethnicity that reported having access to internet at home according to The United States Census Bureau. The data included in this figure are also provided in a table format in Tables S2a and S2b. Individuals who reported having access to the internet, with or without paying a cell phone company or internet service provider, in 2016 (blue), are shown with the increase for each subgroup from 2016 to 2019 in orange and 2019 to 2021 shown in green. The year 2016 is selected as baseline as that is when the DCM Precision Medicine study began enrollment. The years 2019 and 2021 were selected to demonstrate additional increase in access before and after the COVID-19 pandemic. The percentage reporting to have no access at home as of 2021 is shown in yellow and those living in group quarters or housing units vacant at the time of data collection (“N/A”) in 2021 are shown in gray. Generational age groups including generations with individuals ≥18 years of age as of 2023 are presented, including Generation Z (born 2012–1997), Millennials (1996–81), Generation X (1980–65), Baby Boomer (1964–46), and Silent Generation (1945–28). (For interpretation of the references to color in this figure legend, the reader is referred to the web version of this article.)

The DCM Precision Medicine study used a traditional, in-person enrollment model to conduct a family-based study design [8], and while successful, was labor intensive. To achieve the Discovery Study's ambitious enrollment target, novel approaches are required. With the emergence of electronic health portals and applications, successful implementation of digital recruitment and electronic data collection in numerous cardiovascular clinical trials [[16], [17], [18]], and nearly 93 % of American adults reporting access to internet connected devices as of 2021 (Fig. 1; Table S1) [19], there is substantial opportunity to leverage web-based methods. A move to web-based approaches was rated favorably by a majority of sampled DCM Research Project participants and researchers [20].

The DCM Project Portal is a direct-to-participant electronic recruitment, consent, and communication tool designed for family recruitment to the DCM Research Project. The objectives of the portal are to maximize staff efficiency and improve access to participation in The DCM Discovery study, while also facilitating long-term participant engagement. Here, we describe the design and rationale of the DCM Project Portal to conduct family-based genetic research.

## 2 Materials and methods

### 2.1 Design of the DCM Project Portal

The key needs for the design of an electronic approach to recruitment and enrollment were derived from the study's extensive experience with family-based study designs using a traditional, in-person process in prior DCM Research Project investigations (Section 2.3). Most important has been the relationships built with participating families thus far, which have been critical to study success over time. Thus, a central goal for the portal was to serve as a communication interface and to continue to support these important relationships with participating families by providing an open, accessible, and easy communication platform. Other data also contributed to the key needs of the portal design, including experiences of other studies using digital enrollment methods [16,18,[21], [22], [23]], recommended best practices for digital technology use in trials [23], characteristics of the DCM Precision Medicine study participants, US Census-derived estimates of internet access of the US population, and current participant feedback [20] regarding important aspects of web-based models. The following features were prioritized.

The first key feature was a “direct-to-participant” design, meaning that the web-based process can be entirely participant guided. This was prioritized to reduce staff time [18] needed to recruit and enroll each individual in a family unit, increase the accessibility of participation beyond only those that receive care at DCM Consortium sites (Fig. S1), and decrease the burden of unnecessary in person visits [24]. A direct-to-participant approach is also an important strategy to distribute self-administered methods of data collection [18,23].

A second feature important for the portal was to be adaptable to continued growth of the research program and to be able to be tailored to individual user's needs. We anticipate new ancillary studies, greater numbers of requests for families to invite additional relatives to join the study, and greatly expanded data collection needs. To accommodate continued programmatic growth, an adaptable structure allowing for modules or features to be easily added will yield the most benefit and allow the portal to be a long-term resource. For families, similar adaptability is needed at the user level, so that the user experience can be tailored to the needs of participants, such as activation of proband- or family member-participant specific items or allowing users to skip items that do not pertain for a more efficient experience.

A third key design feature was to foster engagement of participants with the research program [23], congruent with the central goal of maintaining long-term relationships with participating families. By engagement we primarily mean maintaining participant-study staff contact to so that participants are able to keep study staff informed of relevant changes to their history. Engagement also includes remaining available, for example, to receive communications regarding new studies they may be eligible for or new data that may need to be collected.

Other important features were focused on operational aspects and prioritized feedback from current study participants, including an advised time to complete the process of 30 minutes or less, having technical support available, and the ability to accommodate varying skillsets [20]. The characteristics of current DCM Precision Medicine study participants were considered as a model participant demographic for user populations for future studies to anticipate potential support and infrastructure needs. Informational text and videos were internally developed by DCM Research Project investigators and personnel. The current version is only available in English with future plans to fully translate this tool to Spanish. However, if an individual indicates that they are Spanish speaking, alternative on-site or telephone-based enrollment processes are available.

A three-phase beta testing process involving participants, study staff, and investigators was completed (Fig. S2). Technical development of The DCM Project Portal was conducted by an external developer under the direction of study personnel. The portal is securely housed at The Ohio Supercomputer Center (OSC) (www.osc.edu) and requires two-factor authentication for login.

### 2.2 Demographic, personal health literacy, and internet usage information

Demographic, personal health literacy, and internet usage information was collected from The DCM Precision Medicine study participants at the time of enrollment. These data were used to consider the design and potential efficacy for a web-based model. Structured interviews collected social and demographic information. Personal health literacy and internet usage characteristics were assessed by six questions in a self-administered survey, inquiring difficulty, confidence, and assistance needs with understanding and completing medical information about themselves in addition to internet accessibility and use for medical purposes. Ages groups were selected based on year of birth by generational classifications. Race-ethnicity was based on self-identification and grouped as non-Hispanic Black, non-Hispanic White, and Hispanic.

To evaluate population internet access, responses to the “Access to Internet” variable, inquiring if individuals have access to internet connected devices at home by paid or unpaid services, was exported from the Public Use Microdata Sample (PUMS) using the US Census Bureau's Microdata Access Tool (MDAT) based on self-identified race, ethnicity, and year of birth by generational classifications. ACS 1-Year Estimates PUMS was selected to generate the data.

### 2.3 Design considerations from the DCM Precision Medicine Study experience

The DCM Precision Medicine study was a multi-site, cross-sectional family-based study executed through the multi-site DCM Consortium led by The Ohio State University (OSU) (Fig. S1) [8]. Institutional Review Boards at OSU and all clinical sites approved the study in the initial period. During the study, a single IRB at the University of Pennsylvania was created with reliance agreements from all clinical sites. Written informed consent was obtained from all participants. The single IRB at the University of Pennsylvania has approved the DCM Project Portal.

Using an in-person model, the study accrued a diverse (nearly half non-Hispanic Black and nearly half female) population including over 1200 probands with idiopathic DCM (clinical criteria provided in Supplemental Material) between June 2016 and March 2020 and over 2000 of their family members through March 2021. Data from participants aged 18 or older were analyzed for the current investigation. Family member recruitment was a multi-step process and required several individuals, staff and participants, as well as substantial oversight to achieve. In short, enrolled probands were asked to refer first degree relatives (parents, siblings, children) and other family member(s) affected with heart disease to the study. With written permission, study staff worked with probands to follow up on referrals and enroll relatives. Data was collected for all participants by medical record review and questionnaires collected by structured interview regarding cardiovascular history, quality of life, health behaviors, family dynamics and family medical history. Of note, enrollment activity was truncated in part by the COVID-19 pandemic due to the limitations on in-person research activities.

## 3 Results

### 3.1 The participant experience

**Fig. 2:**
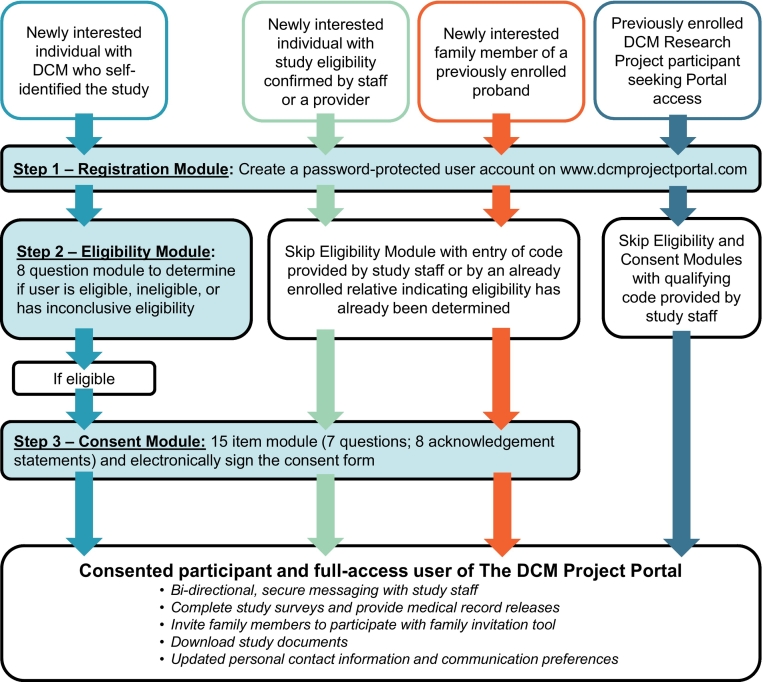
The three step DCM Project Portal process by user type. The three-step process tailored to the user type is shown, including (1) Registration, (2) Eligibility, and (3) Consent Modules. The Registration step establishes an account by collecting basic demographic and contact information, permission to communicate through text messaging, and preferred language. To complete Registration, users are required to create a password so that they may exit and return to their account. If the registered user does not provide skip code indicating that they are eligible, they are directed to the Eligibility Module. This section consists of a series of eight self-guided “yes,” “no,” or “not sure” questions adapted from the study's eligibility criteria (Supplemental Material). Upon completion, logic is built to determine in real time if a user is eligible, ineligible, or has inconclusive eligibility, which requires additional staff review to determine the status. The Consent Module for eligible individuals is similarly built, consisting of a 15-item design with seven questions and eight acknowledgment statements. Upon completion of the Consent Module an electronic signature is collected. Once a user has completed the process specific to their user type, as indicated by the four colored pathways shown, they have full access to the user features of the portal.

The portal is available to previously consented and newly interested individuals. Newly interested individuals may self-discover the study or be referred by a provider or other individual familiar with the research program. A workflow outlining the three-step process tailored to the user type is shown in Fig. 2, including (1) Registration, (2) Eligibility, and (3) Consent Modules. Supporting text (“*Read More*”) and video (“*Watch More*”) resources are available for each item throughout. By current participant advisement [23], users are able to message study staff (“*Ask More*”) at any point. The process is customizable by entering unique, staff-generated codes to allow skipping of sections based on user type, such as indicating that the user has already had eligibility screening, provided consent, or is a family member of a current participant. Upon completion of the Consent Module, the user is shown a full copy of the consent form for final review in REDCap [26], and an electronic signature is collected. A PDF file of the signed form is immediately available for download upon submitting. Electronically formatted surveys and medical record release forms are available once consented, reducing staff time that would typically be required to collect this information.

The Consent Module was designed to prioritize key components of the informed consent process, including accommodation of the dynamic nature of this process and maximizing the autonomy of the research volunteer [[25], [26], [27]]. The simple question-based design, provision of *Read, Watch, and Ask More* resources, and the option to save, exit, and return to modules were put in place to ensure users are adequately informed, not coerced, and understand their choice to participate.

### 3.2 Participant engagement tools

The *Family Invitation* tool is a participant-directed family recruitment tool, where an individual can send a personally signed, pre-written invitation email explaining the study and how to get involved. This digital family recruitment process can occur without staff involvement unless assistance is requested, in contrast to the described previous time intensive process (Section 2.3). In addition, secure messaging is available for users once registered. When a new message is sent, participants receive a text (or email as preferred) notifying them that a message is available, resembling an electronic health record communication model.

### 3.3 The staff interface

The landing page of the staff interface shows a live log of user activity. Staff can access records, correspond, review and update eligibility and consent statuses, download documents, and view if family members have been invited at an individual user level. Staff are given notice if a user has been inactive after three automated reminders for needed information, indicating a need for a follow up phone call. As noted, staff can produce unique codes to validate a user's consent or eligibility status, as applicable, to tailor the user experience. Study surveys and newsletters can be distributed at individual or group-level communications. Communication fields are also built in for staff-to-staff information exchange.

### 3.4 Participant characteristics evaluated for development

Characteristics of participants aged 18 years and older at the time of enrollment to the DCM Precision Medicine Study was evaluated as an exemplary population of anticipated portal users (Table 1). After excluding minors, this included 1223 probands and 1781 first-degree relatives (FDRs) 43.7 % and 60.4 % were female, respectively; 42.2 % probands and 28.3 % of family members identified as non-Hispanic Black, 49.3 % and 62.3 % non-Hispanic white, and 8.5 % and 9.4 % Hispanic. Additional characteristics are summarized in Table 1.

**Table 1:** Characteristics of participants age eighteen and older in the DCM Precision Medicine Study by participant type.

Characteristic	Participant type - No. (%)		
	Total	Proband	Family member
Total	3004 (100 %)	1223 (100 %)	1781 (100 %)
Sex			
Female	1610 (53.6 %)	535 (43.7 %)	1075 (60.4 %)
Male	1394 (46.4 %)	688 (56.3 %)	706 (39.6 %)
Self-reported race-ethnicity group			
Non-Hispanic Black	1014 (34.0 %)	516 (42.2 %)	498 (28.3 %)
Non-Hispanic White	1697 (56.9 %)	602 (49.3 %)	1095 (62.3 %)
Hispanic	270 (9.1 %)	104 (8.5 %)	166 (9.4 %)
Age at enrollment, years			
18–26	347 (11.6 %)	55 (4.5 %)	292 (16.6 %)
27–42	794 (26.6 %)	262 (21.4 %)	532 (30.2 %)
43–58	988 (33.1 %)	516 (42.2 %)	472 (26.8 %)
59–76	777 (26.1 %)	368 (30.1 %)	409 (23.3 %)
≥77	75 (2.5 %)	21 (1.7 %)	54 (3.1 %)
Age at DCM diagnosis, years^a^	N/A^a^	44.2 ± 13.5 years (4.7–82.7)	N/A^a^
Geographic location			
Northeast	348 (11.6 %)	153 (12.5 %)	195 (11.0 %)
Midwest	1040 (34.7 %)	390 (31.9 %)	650 (36.7 %)
South	1101 (36.8 %)	493 (40.3 %)	608 (34.3 %)
West	505 (16.9 %)	186 (15.2 %)	319 (18.0 %)
Education, years			
≤12	1076 (37.8 %)	451 (39.3 %)	625 (36.8 %)
>12	1773 (62.2 %)	698 (60.7 %)	1075 (63.2 %)
Work status			
Employed for wages	1380 (47.1 %)	396 (33.3 %)	984 (56.4 %)
Self-employed	278 (9.5 %)	115 (9.7 %)	163 (9.3 %)
Out of work	271 (9.2 %)	187 (15.7 %)	84 (4.8 %)
A home-maker	133 (4.5 %)	49 (4.1 %)	84 (4.8 %)
A student	108 (3.7 %)	23 (1.9 %)	85 (4.9 %)
Retired	664 (22.6 %)	353 (29.7 %)	311 (17.8 %)
Marital status			
Married	1510 (51.5 %)	650 (54.7 %)	860 (49.3 %)
Divorced	347 (11.8 %)	164 (13.8 %)	183 (10.5 %)
Widowed	114 (3.9 %)	48 (4.0 %)	66 (3.8 %)
Separated	65 (2.2 %)	34 (2.9 %)	31 (1.8 %)
Never married	713 (24.3 %)	224 (18.8 %)	489 (28.0 %)
Member of an unmarried couple	130 (4.4 %)	46 (3.9 %)	84 (4.8 %)

Denominators differ due to inclusion of probands 18 and older and missing or no response values.

a Age at diagnosis only calculated for probands as many family members were not affected with dilated cardiomyopathy at the time of enrollment.

A majority of participants across all age and race-ethnicity groups reported minimal difficulty (*not at all* or *rarely* having problems) learning about their medical condition due to understanding written information and high confidence in filling out medical forms independently (*very much* or *often* feeling confident) (Table 2). While a majority of participants reported having internet access at home, 31.9 % of participants ≥77 years old reported no access (Table 2), though this age group only represented 2.5 % of the DCM Precision Medicine study (Table 1). Other groups with highest reports of no home internet access in included participants who self-identified as non-Hispanic Black (25.2 %) or Hispanic ethnicity (22.9 %) (Table 2), reflecting similar access patterns to that of the general population (Fig. 1). According to the US Census Data the rates of internet access has improved from 2016 to 2021 in these populations (Fig. 1; Tables S1–S5). Additional personal health literacy and internet usage characteristics of participants by age group and self-identified race-ethnicity are shown in Table 2.

**Table 2:** Personal health literacy and internet access and usage characteristics of DCM Precision Medicine study participants by age and race-ethnicity groups.

Questions	Responses	Generational age groups - No. (%)						Self-reported race-ethnicity groups^a^ - No. (%)			
		Total^b^ (N)	18–26	27–42	43–58	59–76	≥77 years	Total^b^	NHE-B^a^	NHE-W^a^	Hispanic
Personal health literacy		N = 2933	N = 342	N = 787	N = 974	N = 758	N = 72	N = 2910	N = 973	N = 1671	N = 266
How often do you have problems learning about your medical condition because of difficulty understanding written information?	Not at all	1562	158 (46.2 %)	444 (56.4 %)	542 (55.6 %)	383 (50.5 %)	35 (48.6 %)	1551	539 (55.4 %)	879 (52.6 %)	133 (50.0)
	Rarely	814	104 (30.4 %)	203 (25.8 %)	259 (26.6 %)	227 (29.9 %)	21 (29.2 %)	804	257 (26.4 %)	483 (28.9 %)	64 (24.1 %)
	Some	376	54 (15.8 %)	100 (12.7 %)	112 (11.5 %)	102 (13.5 %)	8 (11.1 %)	374	102 (10.5 %)	224 (13.4 %)	48 (18.0 %)
	Often	67	9 (2.6 %)	16 (2.0 %)	24 (2.5 %)	13 (1.7 %)	5 (6.9 %)	67	25 (2.6 %)	35 (2.1 %)	7 (2.6 %)
	Very much	43	7 (2.0 %)	12 (1.5 %)	13 (1.3 %)	9 (1.2 %)	2 (2.8 %)	43	20 (2.1 %)	16 (1.0 %)	7 (2.6 %)
How confident are you filling out medical forms by yourself?	Not at all	179	10 (2.9 %)	35 (4.4 %)	59 (6.1 %)	60 (7.9 %)	15 (20.8 %)	179	69 (7.1 %)	81 (4.8 %)	29 (10.9 %)
	Rarely	109	19 (5.6 %)	26 (3.3 %)	41 (4.2 %)	19 (2.5 %)	4 (5.6 %)	108	44 (4.5 %)	52 (3.1 %)	12 (4.5 %)
	Some	315	64 (18.7 %)	62 (7.9 %)	87 (8.9 %)	88 (11.6 %)	14 (19.4 %)	314	87 (8.9 %)	197 (11.8 %)	30 (11.3 %)
	Often	466	107 (31.3 %)	112 (14.2 %)	131 (13.4 %)	103 (13.6 %)	13 (18.1 %)	463	131 (13.5 %)	279 (16.7 %)	53 (19.9 %)
	Very much	1798	131 (38.3 %)	541 (68.7 %)	635 (65.2 %)	465 (61.3 %)	26 (36.1 %)	1780	617 (63.4 %)	1028 (61.5 %)	135 (50.8 %)
How often do you have someone help you read hospital materials?	Not at all	1607	133 (38.9)	483 (61.4 %)	566 (58.1 %)	399 (52.6 %)	26 (36.1 %)	1589	550 (56.5 %)	923 (55.2 %)	116 (43.6 %)
	Rarely	660	107 (31.3 %)	180 (22.9 %)	190 (19.5 %)	163 (21.5 %)	20 (27.8 %)	658	203 (20.9 %)	394 (23.6 %)	61 (22.9 %)
	Some	337	52 (15.2 %)	74 (9.4 %)	109 (11.2 %)	92 (12.1 %)	10 (13.9 %)	336	95 (9.8 %)	199 (11.9 %)	42 (15.8 %)
	Often	147	28 (8.2 %)	23 (2.9 %)	45 (4.6 %)	45 (5.9 %)	6 (8.3 %)	145	50 (5.1 %)	70 (4.2 %)	25 (9.4 %)
	Very much	104	9 (2.6 %)	16 (2.0 %)	37 (3.8 %)	34 (4.5 %)	8 (11.1 %)	104	40 (4.1 %)	50 (3.0 %)	14 (5.3 %)
Internet access and usage		N = 2933	N = 342	N = 787	N = 974	N = 758	N = 72	N = 2910	N = 973	N = 1671	N = 266
Do you have internet access at home?	Yes^c^	2422	279 (81.6 %)	661 (84.0 %)	810 (83.2 %)	625 (82.5 %)	47 (65.3 %)	2400	705 (72.5 %)	1499 (89.7 %)	196 (73.7 %)
	No	445	55 (16.1 %)	115 (14.6 %)	143 (14.7 %)	109 (14.4 %)	23 (31.9 %)	444	245 (25.2 %)	138 (8.3 %)	61 (22.9 %)
In the past 12 months, did you use the internet, whether from home or somewhere else to look for health or medical information for yourself?	Yes	2213	239 (69.9 %)	630 (80.1 %)	744 (76.4 %)	561 (74.0 %)	39 (54.2 %)	2196	695 (71.4 %)	1326 (79.4 %)	175 (65.8 %)
	No	632	94 (27.5 %)	142 (18.0 %)	199 (20.4 %)	167 (22.0 %)	30 (41.7 %)	626	241 (24.8 %)	307 (18.4 %)	78 (29.3 %)
In the past 12 months, how often did you use the internet to look for health or medical information for yourself or someone else?	Not Applicable	13	3 (0.9 %)	0	3 (0.3 %)	5 (0.7 %)	2 (2.8 %)	13	4 (0.4 %)	9 (0.5 %)	0 (0.0 %)
	Once a week	635	58 (17.0 %)	196 (24.9 %)	226 (23.2 %)	146 (19.3 %)	9 (12.5 %)	630	236 (24.3 %)	321 (19.2 %)	73 (27.4 %)
	Once a month	633	69 (20.2 %)	180 (22.9 %)	228 (23.4 %)	151 (19.9 %)	5 (6.9 %)	629	198 (20.3 %)	385 (23.0 %)	46 (17.3 %)
	Every few months	693	77 (22.5 %)	200 (25.4 %)	222 (22.8 %)	179 (23.6 %)	15 (20.8 %)	685	194 (19.9 %)	439 (26.3 %)	52 (19.5 %)
	Less often than every few months	850	123 (36.0 %)	194 (24.7 %)	267 (27.4 %)	233 (30.7 %)	33 (45.8 %)	844	300 (30.8 %)	466 (27.9 %)	78 (29.3 %)

a NHE-B = non-Hispanic Black; NHE-W = non-Hispanic White.

b The number of total responses differs from the total counts of individuals in each demographic group shown in Table 1 due to missing responses.

c Individuals who marked “yes” to having internet access at home include those who had access through their phone, dialup, and/or broadband or highspeed internet.

## 4 Discussion

The DCM Project Portal to our knowledge is the first multi-faceted, web-based tool developed to support family-based genetic investigations of DCM. As described, the portal is designed to facilitate the enrollment of large numbers of patients with DCM and their family members and has been approved by the DCM Research Project's single IRB. Beta testing has been accomplished and the portal has now entered service.

Family units are a critical scientific need for investigations of The DCM Research Project. However, working with families adds to recruitment complexities. It has been observed across families with shared genetic disease risk that information sharing can be selective [[28], [29], [30]] and several barriers to communicating about genetic risk information have been described in families, including emotional, geographic, health literacy, self-efficacy, guilt, and others, all of which can make recruitment of a family unit, rather than an individual patient, more challenging [28,29]. Incomplete participation of family members at risk for genetic cardiovascular disease has been observed by The DCM Research Project [10] and others [31,32]. Offering multiple modalities, including non-web-based options, and simple processes to engage one's family has the potential to help bypass communication barriers and improve participation of family units.

Direct-to-participant digital recruitment and enrollment modalities are becoming increasingly used to conduct clinical research [16,18,[21], [22], [23]]. As the population internet usage continues to increase (Fig. 1; Tables S1–S5), the integration of electronic strategies will be needed to achieve large enrollment goals, with potential to capture more individuals and maximize efficiency of study staff time. However, the development of digital platforms requires extensive effort and must be tailored to unique needs of a specific study or research program [16,23].

Diversity in genetic and genomic research is essential, and the use of multiple enrollment modalities may also be critical to support diversity of participants [21]. Although US Census data suggests internet access has increased since 2016 across generational age and race-ethnicity groups (Fig. 1), possibly in part due to the shift toward digital environments as a result of the COVID-19 pandemic, individuals in the population who are self-identified black, Hispanic, and were born between the years of 1946–1964 (Baby boomer generation) or 1928–1945 (Silent Generation), had the highest reported rates of not having access to the internet at their home in 2021 (6.6 %, 5.1 %, 7.6 %, and 18.2 %, respectively; Fig. 1; Tables S1–S5). A similar pattern as was reported by study participants identifying as non-Hispanic Black, Hispanic, and over the age of 77 years (Table 2). The rates of no access were higher in participants in these groups compared to the proportions reported in the population, however data were collected from study participants at the time of enrollment which could be as early as 2016 when connectivity rates were lower in general. To remedy this issue and to maximize access and support participants of diverse backgrounds, web- and non-web options will be available, as has also been done by others [22] and is a need emphasized by study participants [20]. The developed portal described herein will not replace established in-person or telephone-based options for enrollment but extend enrollment opportunities by adding an additional web-based model for those who are able.

While initial beta testing has been completed (Fig. S2), opportunities for improvement are anticipated. Specifically, we will monitor the self-guided, question based Eligibility Module performance, as when previously surveyed current participants and researchers were asked, “*How confident are you in [your/your patient's] ability to accurately answer questions about your eligibility for The DCM Research Project by answering a series of questions about your cardiovascular health history*,” only 56 % of DCM Project researchers were confident or somewhat confident participants could do so, contrasting with a high level of confidence (87 % confident or somewhat confident) reported by the sampled participants [20]. A similar high confidence level and lack of difficulty in understanding and reporting one's medical information was also reported by the larger set of DCM Precision Medicine Study participants (Table 2). Whether perceived confidence and lack of difficulty will result in accuracy of responses is unknown [23]. Piloting of surveys will be necessary prior to portal integration to optimize digital data collection as instruments are added to the tool. With the addition of new surveys or the communication of more complex information through the study, such as returning genetic information, integrating comprehension questionnaires may also be needed to evaluate understanding. The supportive *Read More*, *Watch More*, and *Ask More* features were developed in the current version to help mitigate this potential challenge that will be closely evaluated with increasing participant accrual.

An additional area for monitoring is potential risk for loss to follow up with a web-initiated approach, which could limit the development of a personal relationship with a participant [23]. As an initial preventive measure, automated message reminders connected with text message alerts are issued if modules are incomplete or if they have a new message. The text-based communication eliminates the participant need to independently remember to return to the portal by actively engaging with them. Text messaging approaches have also been successful in other trials in improving medication adherence [33]. Other strategies may be required if loss to follow up is observed at an undesirable rate.

Beyond anticipated challenges, we look forward to areas for future innovation, such as a method for administration of randomized interventions. Having participants consented for recontact and electronically accessible also enables easy-access and low-pressure recruitment pathways to new study and trial opportunities. Although optimization of digital approaches can be tedious to develop and maintain, the potential positive impact on participant access and efficiency of research operations presents substantial opportunity for studies seeking large sample sizes and long-term engagement.

### 4.1 Limitations

The portal was designed to facilitate the needs of a family-based genetic study for DCM, and therefore the design was developed to address the aims specific to that topic and may not be generalizable for other uses. In addition, only people of self-identified Black, White, and Hispanic race-ethnicity groups enrolled from 2016 to 2020 using a traditional, face-to-face enrollment model were included, who overall rated electronic methods favorably, which may not be representative of individuals who identify with other race-ethnicity groups or those without access to in-person research enrollment methods.

## 5 Conclusions

The DCM Project Portal is a web-based, direct-to-participant platform tailored to the DCM Research Project. A similar web-based design may be useful for other clinical genetics and family-based studies. Customization of the framework reported herein to other programs with similar needs has the potential to augment traditional processes.

## CRediT authorship contribution statement

**Elizabeth S. Jordan:** Conceptualization, Data curation, Formal analysis, Investigation, Methodology, Project administration, Supervision, Validation, Visualization, Writing – original draft, Writing – review & editing. **Phoenix L. Grover:** Investigation, Project administration, Writing – original draft, Writing – review & editing. **Jay Lin:** Data curation, Formal analysis, Investigation, Methodology, Project administration, Supervision, Validation, Visualization, Writing – original draft, Writing – review & editing. **Carl A. Starkey:** Conceptualization, Supervision. **Elizabeth A. Finley:** Conceptualization, Project administration, Supervision. **Hanyu Ni:** Data curation, Formal analysis, Supervision. **Ray E. Hershberger:** Conceptualization, Formal analysis, Funding acquisition, Investigation, Methodology, Project administration, Resources, Supervision, Visualization, Writing – original draft, Writing – review & editing.

## Declaration of competing interest

The authors declare that they have no known competing financial interests or personal relationships that could have appeared to influence the work reported in this paper.

## Appendix A Supplementary data

Supplementary material Image 1
