# Supplementary material for: The DCM Project Portal: A direct-to-participant platform of The DCM Research Project
Source: Am Heart J Plus. Author manuscript; Available in PMC 2024 Feb 12. (PMC10861184; doi:10.1016/j.ahjo.2023.100356)
Supplement: 1 [file NIHMS1964412-supplement-1.docx]

**Supplemental Material**

Inclusion/Exclusion Criteria for probands in the DCM Precision Medicine Study

All probands have met diagnostic criteria for idiopathic DCM: left ventricular ejection fraction <50%; left ventricular enlargement (echo-derived left ventricular end-diastolic dimension ≥95th percentile for gender/height). In addition, they were in one of the target ethnicity-ancestry groups, were able to communicate in English (or in Spanish at sites approved to recruit individuals of Hispanic ethnicity), were able to give informed consent (or assent and parental consent for children), and were willing and able to participate in a family-based study. Relatives also were expected to satisfy these same requirements except for meeting diagnostic criteria for idiopathic DCM. Risk factors considered conventional for DCM, such as obesity, routinely treated hypertension, alcohol use/abuse, peripartum cardiomyopathy, or the presence of left-ventricular non-compaction, were not used as exclusion criteria.

Exclusion criteria included: Coronary artery disease (CAD) causing ischemic cardiomyopathy (>50% narrowing, any major epicardial coronary artery; clinical testing is indicated to exclude CAD routinely in (1) males >40 years or females >45 years without risk factors and (2) males or females >30 years with risk factors); Primary valvular disease; Cardiotoxic drug exposure, including Adriamycin and other cancer chemotherapeutics; Other forms of cardiomyopathy (e.g., hypertrophic cardiomyopathy, arrhythmogenic right ventricular cardiomyopathy, restrictive cardiomyopathy, Chagas cardiomyopathy); Congenital/structural heart disease; Sarcoid; Amyloid; Iron overload; Other active multisystem disease that may plausibly cause DCM (e.g., hypereosinophilic syndrome, cardiac involvement with connective tissue disease, Loeffler’s endocarditis, endomyocardial fibrosis); Severe and untreated or untreatable hypertension (defined as systolic blood pressures routinely >180 mm Hg and/or diastolic blood pressures >120 mm Hg. Untreated hypertension is an individual receiving no medications, and untreatable hypertension is an individual with severe hypertension persisting despite multidrug regimens and/or associated with other multisystem disease (e.g., scleroderma, other vasculitides, etc)); Exclusion beyond a reasonable doubt of all other detectable causes of cardiomyopathy (other than genetic) at the time of primary DCM diagnosis using a rigorous clinical standard congruent with an expert approach rendered by a board-certified heart failure/transplant cardiologist.

**Tables S1-S5. Reported access to the internet in the United States population by generational age group and self-identified race and Hispanic ethnicity**

To estimate internet connectivity in the population by self-identified race, ethnicity, generational age groups, data was exported from the Public Use Microdata Sample (PUMS) using the US Census Bureau’s Microdata Access Tool (MDAT). ACS 1-Year Estimates PUMS was selected with the corresponding year to generate the dataset. Tables were generated by selecting the “Access to Internet” variable and the aforementioned demographic variables. “Age” was customized to captured to encompass age groups defined by birth years within generations (Generation Z: 1997-2012, Millennials: 1981-86, Generation X: 1965-80, Baby Boomers: 1946-64, Silent Generation: 1928-45, WWII generation: 1901-1927). Data were exported for each variable combination for 2016, 2019, and 2021 to determine if there was a reported increase over the years, in part as a possible impact of the COVID-19 pandemic on internet connectivity.

**Table S1. Total ACS 1-Year Estimates Access to the Internet in 2019 and 2021 According to the Public Use Microdata Sample**

| **Access to the Internet in the United States (2019)** | | | |
| --- | --- | --- | --- |
|  | 2016 | 2019 | 2021 |
| *N/A*^a^ | 8079879 (2.5%) | 8084362 (2.5%) | 7760859 (2.3%) |
| *Yes, Total (with or without paying a service^b^)* | 280016149 (86.7%) | 295581562 (90.0%) | 308066247 (92.8%) |
| *Yes, by paying a service* | 271175590 (83.9%) | 288656692 (87.9%) | 301737502 (90.9%) |
| *Yes, without paying a service* | 8840559 (2.7%) | 6924870 (2.1%) | 6328745 (1.9%) |
| *No access*^c^ | 35031487 (10.8%) | 24573599 (7.5%) | 16066639 (4.8%) |
| **Total** | 323127515 (100%) | 328239523 (100%) | 331893745 (100%) |

^a^N/A refers to individuals who lived in group quarters or housing units that were vacant at the time of data collection

^b^service refers to paying a cell phone company of internet service provider

^c^No responses are regarding to access at home; may not be representative of access to public internet sources

**Table S2a. Reported Internet Access in the US Population in 2016 and 2021 According to Census Data**

|  | **2016 (%)** | | | | | **2021 (%)** | | | | |  |
| --- | --- | --- | --- | --- | --- | --- | --- | --- | --- | --- | --- |
|  | **Yes, Total (with or without paying a service)** | **Yes, by paying a service^b^** | **Yes, without paying a service** | **No Access^c^** | **N/A^d^** | **Yes, Total (with or without paying a service)** | **Yes, by paying a service^b^** | **Yes, without paying a service** | **No Access^c^** | **N/A^d^** | **Change in Reported Internet Access between 2016 and 2021** |
| **Generational Age Groups^a^** (generation name, corresponding years of birth listed) |  |  |  |  |  |  |  |  |  |  |  |
| Generation Z (2012-1997) | 89.52% | 86.85% | 2.66% | 7.68% | 2.80% | 92.68% | 90.74% | 1.94% | 2.41% | 4.91% | +2.80% |
| Millennials (1996-1981) | 88.73% | 85.68% | 3.05% | 7.49% | 3.78% | 95.28% | 93.62% | 1.65% | 2.68% | 2.04% | +3.78% |
| Generation X (1980-1965) | 90.25% | 87.67% | 2.58% | 8.04% | 1.71% | 94.88% | 93.16% | 1.72% | 3.60% | 1.52% | +1.71% |
| Baby Boomer (1964-1946) | 85.92% | 83.40% | 2.52% | 12.74% | 1.35% | 91.06% | 88.98% | 2.08% | 7.56% | 1.38% | +1.35% |
| Silent Generation  (1945-1928) | 69.85% | 66.93% | 2.92% | 26.96% | 3.19% | 77.91% | 74.79% | 3.12% | 18.16% | 3.92% | +3.19% |
| **Self-identified Race** |  |  |  |  |  |  |  |  |  |  |  |
| Black | 78.81% | 75.17% | 3.64% | 16.82% | 4.37% | 89.03% | 86.54% | 2.50% | 6.64% | 4.32% | +10.22% |
| White | 87.73% | 85.15% | 2.58% | 10.02% | 2.25% | 93.09% | 91.30% | 1.78% | 4.66% | 2.25% | +5.36% |
| **Self-identified Ethnicity** |  |  |  |  |  |  |  |  |  |  |  |
| Non-Hispanic | 87.34% | 84.69% | 2.65% | 10.03% | 2.64% | 92.71% | 90.86% | 1.86% | 4.78% | 2.51% | +5.38% |
| Hispanic | 83.52% | 80.36% | 3.16% | 14.61% | 1.87% | 93.28% | 91.16% | 2.12% | 5.11% | 1.61% | +9.76% |

^a^only showing generations including those aged >18 years as of 2023 sorted by generational name and years of birth.

^b^service refers to paying a cell phone company of internet service provider

^c^No responses are regarding to access at home; may not be representative of access to public internet sources

^d^N/A refers to individuals who lived in group quarters or housing units that were vacant at the time of data collection

**Table S2b. Reported Internet Access in the US Population in 2019 and 2021 According to Census Data**

|  | **2019 (%)** | | | | | **2021 (%)** | | | | |  |
| --- | --- | --- | --- | --- | --- | --- | --- | --- | --- | --- | --- |
|  | **Yes, Total (with or without paying a service)** | **Yes, by paying a service^b^** | **Yes, without paying a service** | **No Access^c^** | **N/A^d^** | **Yes, Total (with or without paying a service)** | **Yes, by paying a service^b^** | **Yes, without paying a service** | **No Access^c^** | **N/A^d^** | **Change in Reported Internet Access between 2019 and 2021** |
| **Generational Age Groups^a^** (generation name, corresponding years of birth listed) |  |  |  |  |  |  |  |  |  |  |  |
| Generation Z (2012-1997) | 90.69% | 88.53% | 2.16% | 4.54% | 4.77% | 92.68% | 90.74% | 1.94% | 2.41% | 4.91% | +1.99% |
| Millennials (1996-1981) | 93.03% | 91.02% | 2.01% | 4.67% | 2.30% | 95.28% | 93.62% | 1.65% | 2.68% | 2.04% | +2.24% |
| Generation X (1980-1965) | 92.88% | 91.03% | 1.85% | 5.49% | 1.63% | 94.88% | 93.16% | 1.72% | 3.60% | 1.52% | +2.00% |
| Baby Boomer (1964-1946) | 88.58% | 86.46% | 2.12% | 9.96% | 1.46% | 91.06% | 88.98% | 2.08% | 7.56% | 1.38% | +2.48% |
| Silent Generation  (1945-1928) | 73.72% | 70.72% | 2.99% | 22.75% | 3.53% | 77.91% | 74.79% | 3.12% | 18.16% | 3.92% | +4.20% |
| **Self-identified Race** |  |  |  |  |  |  |  |  |  |  |  |
| Black | 84.35% | 81.44% | 2.91% | 11.36% | 4.29% | 89.03% | 86.54% | 2.50% | 6.64% | 4.32% | +4.68% |
| White | 90.80% | 88.77% | 2.03% | 6.99% | 2.21% | 93.09% | 91.30% | 1.78% | 4.66% | 2.25% | +2.29% |
| **Self-identified Ethnicity** |  |  |  |  |  |  |  |  |  |  |  |
| Non-Hispanic | 90.28% | 88.22% | 2.06% | 7.12% | 2.60% | 92.71% | 90.86% | 1.86% | 4.78% | 2.51% | +2.43% |
| Hispanic | 89.02% | 86.69% | 2.33% | 9.12% | 1.87% | 93.28% | 91.16% | 2.12% | 5.11% | 1.61% | +4.26% |

^a^only showing generations including those aged >18 years as of 2023 sorted by generational name and years of birth.

^b^service refers to paying a cell phone company of internet service provider

^c^No responses are regarding to access at home; may not be representative of access to public internet sources

^d^N/A refers to individuals who lived in group quarters or housing units that were vacant at the time of data collection

**Table S3a. ACS 1-Year Estimates Access to the Internet in 2016 According to the Public Use Microdata Sample by Generational Age Group**

| **2016 Internet Usage by Generational Cohorts** | | | | | |
| --- | --- | --- | --- | --- | --- |
| **Source: ACS 1-Year Estimates Public Use Microdata Sample 2016** | | | | | |
| Weight used: PWGTP | | | | | |
| **Access to the Internet (ACCESS)** | | | | | |
| **Generational Age Groups^a^** (generation name, corresponding years of birth listed) | Total | N/A^b^ | Yes, by paying a service^c^ | Yes, without paying a service | No access^d^ |
| **Total^a^** | 323127515 | 8079879 (2.50%) | 271175590 (83.92%) | 8840559 (2.74%) | 35031487 (10.84%) |
| **Generation Z (2012-1997)** | 70386618 | 1972931 (2.80%) | 61133269 (86.85%) | 1875801 (2.66%) | 5403617 (7.68%) |
| **Millennials (1996-1981)** | 71084211 | 2690229 (3.78%) | 60905065 (85.68%) | 2167514 (3.05%) | 5321403 (7.49%) |
| **Generation X (1980-1965)** | 65887779 | 1127419 (1.71%) | 57762154 (87.67%) | 1698933 (2.58%) | 5299273 (8.04%) |
| **Baby Boomer (1964-1946)** | 74037754 | 995980 (1.35%) | 61747186 (83.40%) | 1862654 (2.52%) | 9431934 (12.74%) |
| **Silent Generation (1945-1928)** | 26802746 | 855909 (3.19%) | 17938275 (66.93%) | 783055 (2.92%) | 7225507 (26.96%) |
| **WWII (1901-1924)^e^** | 3005109 | 429574 (14.29%) | 1212812 (40.36%) | 116293 (3.87%) | 1246430 (41.48%) |

^a^only showing generations including those aged >18 years as of 2023 sorted by generational name and years of birth. Total also includes the younger Generation Alpha which does not include individuals aged >18 years as of 2023.

^b^N/A refers to individuals who lived in group quarters or housing units that were vacant at the time of data collection

^c^service refers to paying a cell phone company of internet service provider

^d^No responses are regarding to access at home; may not be representative of access to public internet sources

**^e^**99 is the upper age limit of the Census data and not reflective of the actual age cutoff of this **Table S3b. ACS 1-Year Estimates Access to the Internet in 2019 According to the Public Use Microdata Sample by Generational Age Group**

| **2019 Internet Usage by Generational Cohorts** | | | | | |
| --- | --- | --- | --- | --- | --- |
| **Source: ACS 1-Year Estimates Public Use Microdata Sample 2019** | | | | | |
| Weight used: PWGTP | | | | | |
| **Access to the Internet (ACCESS)** | | | | | |
| **Generational Age Groups^a^** (generation name, corresponding years of birth listed) | Total | N/A^b^ | Yes, by paying a service^c^ | Yes, without paying a service | No access^d^ |
| **Total^a^** | 328239523 | 8084362 (2.46%) | 288656692 (87.94%) | 6924870 (2.11%) | 24573599 (7.49%) |
| **Generation Z (2012-1997)** | 67805245 | 3231114 (4.77%) | 60026497 (88.53%) | 1465901 (2.16%) | 3081733 (4.54%) |
| **Millennials (1996-1981)** | 71525288 | 1641951 (2.30%) | 65104337 (91.02%) | 1438565 (2.01%) | 3340435 (4.67%) |
| **Generation X (1980-1965)** | 65308606 | 1063134 (1.63%) | 59447159 (91.03%) | 1210772 (1.85%) | 3587541 (5.49%) |
| **Baby Boomer (1964-1946)** | 71803346 | 1047398 (1.46%) | 62078234 (86.46%) | 1523892 (2.12%) | 7153822 (9.96%) |
| **Silent Generation (1945-1928)** | 22850856 | 806891 (3.53%) | 16160835 (70.72%) | 683925 (2.99%) | 5199205 (22.75%) |
| **WWII (1901-1924)^e^** | 1880765 | 278439 (14.8%) | 862494 (45.86%) | 85710 (4.56%) | 654122 (34.78%) |

^a^only showing generations including those aged >18 years as of 2023 sorted by generational name and years of birth. Total also includes the younger Generation Alpha which does not include individuals aged >18 years as of 2023.

^b^N/A refers to individuals who lived in group quarters or housing units that were vacant at the time of data collection

^c^service refers to paying a cell phone company of internet service provider

^d^No responses are regarding to access at home; may not be representative of access to public internet sources

**^e^**99 is the upper age limit of the Census data and not reflective of the actual age cutoff of this generation

**Table S3c. ACS 1-Year Estimates Access to the Internet in 2021 According to the Public Use Microdata Sample by Generational Age Group**

| **2021 Internet Usage by Generational Cohorts** | | | | | |
| --- | --- | --- | --- | --- | --- |
| **Source: ACS 1-Year Estimates Public Use Microdata Sample 2021** | | | | | |
| Weight used: PWGTP | | | | | |
| **Access to the Internet (ACCESSINET)** | | | | | |
| **Generational Age Groups^a^** (generation name, corresponding years of birth listed) | Total | N/A^b^ | Yes, by paying a service^c^ | Yes, without paying a service | No access^d^ |
| **Total^a^** | 331893745 | 7760859 (2.34%) | 301737502 (90.91%) | 6328745 (1.91%) | 16066639 (4.84%) |
| **Generation Z (2012-1997)** | 69075253 | 3394180 (4.91%) | 62680388 (90.74%) | 1339025 (1.94%) | 1661660 (2.41%) |
| **Millennials (1996-1981)** | 72109485 | 1471669 (2.04%) | 67512342 (93.62%) | 1191720 (1.65%) | 1933754 (2.68%) |
| **Generation X (1980-1965)** | 65748113 | 1000636 (1.52%) | 61252802 (93.16%) | 1127977 (1.72%) | 2366698 (3.60%) |
| **Baby Boomer (1964-1946)** | 70571030 | 975402 (1.38%) | 62796417 (88.98%) | 1466078 (2.08%) | 5333133 (7.56%) |
| **Silent Generation (1945-1928)** | 18692329 | 733138 (3.92%) | 13980278 (74.79%) | 583471 (3.12%) | 3395442 (18.16%) |
| **WWII (1901-1924)^e^** | 1168943 | 164797 (14.1%) | 611421 (52.31%) | 57470 (4.92%) | 335255 (28.68%) |

^a^only showing generations including those aged >18 years as of 2023 sorted by generational name and years of birth. Total also includes the younger Generation Alpha which does not include individuals aged >18 years as of 2023.

^b^N/A refers to individuals who lived in group quarters or housing units that were vacant at the time of data collection

^c^service refers to paying a cell phone company of internet service provider

^d^No responses are regarding to access at home; may not be representative of access to public internet sources

**^e^**99 is the upper age limit of the Census data and not reflective of the actual age cutoff of this generation

**Table S4a. ACS 1-Year Estimates Access to the Internet in 2016 According to the Public Use Microdata Sample by Self-identified Race**

| **2016 Internet Usage by Race** | | | | | |
| --- | --- | --- | --- | --- | --- |
| **Source: ACS 1-Year Estimates Public Use Microdata Sample 2016** | | | | | |
| Weight used: PWGTP | | | | | |
| **Access to the Internet (ACCESS)** | | | | | |
| **Recoded detailed race code** | Total | N/A^a^ | Yes, by paying a service^b^ | Yes, without paying a service | No access^c^ |
| Total | 323127515 | 8079879 (2.50%) | 271175590 (83.92%) | 8840559 (2.74%) | 35031487 (10.84%) |
| White | 234627780 | 5284931 (2.25%) | 199783840 (85.15%) | 6059702 (2.58%) | 23499307 (10.02%) |
| Black or African American | 40954190 | 1787672 (4.37%) | 30785201 (75.17%) | 1491636 (3.64%) | 6889681 (16.82%) |
| American Indian | 2124701 | 58249 (2.74%) | 1487479 (70.01%) | 77204 (3.63%) | 501769 (23.62%) |
| Alaska Native | 123204 | 3334 (2.71%) | 93083 (75.55%) | 2031 (1.65%) | 24756 (20.09%) |
| American Indian and Alaska Native tribes specified; or American Indian or Alaska Native, not specified and no other races | 429074 | 19390 (4.52%) | 305453 (71.19%) | 16005 (3.73%) | 88226 (20.56%) |
| Asian | 17577855 | 350857 (2.00%) | 16001392 (91.03%) | 371284 (2.11%) | 854322 (4.86%) |
| Native Hawaiian and Other Pacific Islander | 591268 | 19761 (3.34%) | 477314 (80.73%) | 21144 (3.58%) | 73049 (12.35%) |
| Some other race | 16363098 | 267414 (1.63%) | 13173589 (80.51%) | 536517 (3.28%) | 2385578 (14.58%) |
| Two or More Races | 10336345 | 288271 (2.79%) | 9068239 (87.73%) | 265036 (2.56%) | 714799 (6.92%) |

^a^N/A refers to individuals who lived in group quarters or housing units that were vacant at the time of data collection

^b^service refers to paying a cell phone company of internet service provider

^c^No responses are regarding to access at home; may not be representative of access to public internet sources

**Table S4b. ACS 1-Year Estimates Access to the Internet in 2019 According to the Public Use Microdata Sample by Self-identified Race**

| **2019 Internet Usage by Race** | | | | | |
| --- | --- | --- | --- | --- | --- |
| **Source: ACS 1-Year Estimates Public Use Microdata Sample 2019** | | | | | |
| Weight used: PWGTP | | | | | |
| **Access to the Internet (ACCESS)** | | | | | |
| **Recoded detailed race code** | Total | N/A^a^ | Yes, by paying a service^b^ | Yes, without paying a service | No access^c^ |
| Total | 328239523 | 8084362 (2.46%) | 288656692 (87.94%) | 6924870 (2.11%) | 24573599 (7.49%) |
| White | 236532194 | 5233723 (2.21%) | 209980189 (88.77%) | 4794213 (2.03%) | 16524069 (6.99%) |
| Black or African American | 42043031 | 1802226 (4.29%) | 34241467 (81.44%) | 1223914 (2.91%) | 4775424 (11.36%) |
| American Indian | 2248236 | 66416 (2.95%) | 1718308 (76.43%) | 64888 (2.89%) | 398624 (17.73%) |
| Alaska Native | 112956 | 3611 (3.20%) | 90278 (79.92%) | 1199 (1.06%) | 17868 (15.82%) |
| American Indian and Alaska Native tribes specified; or American Indian or Alaska Native, not specified and no other races | 480428 | 29074 (6.05%) | 363711 (75.71%) | 11995 (2.50%) | 75648 (15.75%) |
| Asian | 18608918 | 369549 (1.99%) | 17384042 (93.42%) | 243401 (1.31%) | 611926 (3.29%) |
| Native Hawaiian and Other Pacific Islander | 610822 | 21488 (3.52%) | 520773 (85.26%) | 12008 (1.97%) | 56553 (9.26%) |
| Some other race | 16266146 | 284217 (1.75%) | 14005776 (86.1%) | 367539 (2.26%) | 1608614 (9.89%) |
| Two or More Races | 11336792 | 274058 (2.42%) | 10352148 (91.31%) | 205713 (1.81%) | 504873 (4.45%) |

^a^N/A refers to individuals who lived in group quarters or housing units that were vacant at the time of data collection

^b^service refers to paying a cell phone company of internet service provider

^c^No responses are regarding to access at home; may not be representative of access to public internet sources

**Table S4c. ACS 1-Year Estimates Access to the Internet in 2021 According to the Public Use Microdata Sample by Self-identified Race**

| **2021 Internet Usage by Race** | | | | | | |
| --- | --- | --- | --- | --- | --- | --- |
| **Source: ACS 1-Year Estimates Public Use Microdata Sample 2021** | | | | | | |
| Weight used: PWGTP | | | | | | |
| **Access to the Internet (ACCESSINET)** | | | | | | |
| **Recoded detailed race code** | Total | N/A^a^ | Yes, by paying a service^b^ | Yes, without paying a service | No access^c^ |  |
| Total | 331893745 | 7760859 (2.34%) | 301737502 (90.91%) | 6328745 (1.91%) | 16066639 (4.84%) |  |
| White | 202954389 | 4570818 (2.25%) | 185306842 (91.30%) | 3620575 (1.78%) | 9456154 (4.66%) |  |
| Black or African American | 40217512 | 1739001 (4.32%) | 34802991 (86.54%) | 1003596 (3.50%) | 2671924 (6.64%) |  |
| American Indian | 2562058 | 44465 (1.74%) | 2193725 (85.62%) | 81593 (3.18%) | 242275 (9.46%) |  |
| Alaska Native | 124111 | 5162 (4.16%) | 102668 (82.72%) | 3110 (2.51%) | 13171 (10.61%) |  |
| American Indian and Alaska Native tribes specified; or American Indian or Alaska Native, not specified and no other races | 475644 | 37044 (7.79%) | 384921 (80.93%) | 13027 (2.74%) | 40652 (8.55%) |  |
| Asian alone | 19174507 | 330048 (1.72%) | 18196497 (94.90%) | 236779 (1.23%) | 411183 (2.14%) |  |
| Native Hawaiian and Other Pacific Islander | 623315 | 17319 (2.78%) | 556979 (89.36%) | 20497 (3.29%) | 28520 (4.58%) |  |
| Some other race | 23899490 | 330223 (1.38%) | 21666481 (90.66%) | 548625 (2.30%) | 1354161 (5.67%) |  |
| Two or More Races | 41862719 | 686779 (1.64%) | 38526398 (92.03%) | 800943 (1.91%) | 1848599 (4.42%) |  |

^a^N/A refers to individuals who lived in group quarters or housing units that were vacant at the time of data collection

^b^service refers to paying a cell phone company of internet service provider

^c^No responses are regarding to access at home; may not be representative of access to public internet sources

**Table S5a. ACS 1-Year Estimates Access to the Internet in 2016 According to the Public Use Microdata Sample by Hispanic Ethnicity**

|  |  |  |  |  |  |
| --- | --- | --- | --- | --- | --- |
| **Source: ACS 1-Year Estimates Public Use Microdata Sample 2016** | | | | | |
| Weight used: PWGTP | | | | | |
| **Access to the Internet (ACCESS)** | | | | | |
| **Recoded detailed Hispanic origin** | Total | N/A^a^ | Yes, by paying a service^b^ | Yes, without paying a service | No access^c^ |
| **-> Total** | 323127515 | 8079879 (2.50%) | 271175590 (83.92%) | 8840559 (2.74%) | 35031487 (10.84%) |
| **Not Spanish/Hispanic/Latino** | 265737764 | 7004631 (2.64%) | 225054590 (84.69%) | 7029891 (2.65%) | 26648652 (10.03%) |
| **Total Spanish/Hispanic/Latino^4^** | 57389751 | 1075248 (1.87%) | 46121000 (80.36%) | 1810668 (3.16%) | 8382835 (14.61%) |
| **Mexican** | 36203500 | 596838 (1.65%) | 28834404 (79.65%) | 1096684 (3.03%) | 5675574 (15.68%) |
| **Puerto Rican** | 5506037 | 156773 (2.85%) | 4493679 (81.61%) | 198093 (3.60%) | 657492 (11.94%) |
| **Cuban** | 2204591 | 41133 (1.87%) | 1799538 (81.63%) | 76799 (3.48%) | 287121 (13.02%) |
| **Dominican** | 1914302 | 27990 (1.46%) | 1570398 (82.04%) | 64957 (3.39%) | 250957 (13.11%) |
| **Costa Rican** | 141233 | 1923 (1.36%) | 125313 (88.73%) | 5451 (3.86%) | 8546 (6.05%) |
| **Guatemalan** | 1413205 | 20278 (1.43%) | 1041314 (73.68%) | 52286 (3.70%) | 299327 (21.18%) |
| **Honduran** | 973974 | 15733 (1.62%) | 717964 (73.71%) | 40989 (4.21%) | 199288 (20.46%) |
| **Nicaraguan** | 412914 | 4954 (1.20%) | 355640 (86.13%) | 10610 (2.57%) | 41710 (10.10%) |
| **Panamanian** | 184021 | 7891 (4.29%) | 159018 (86.41%) | 5250 (2.85%) | 11862 (6.45%) |
| **Salvadoran** | 2171302 | 23635 (1.09%) | 1746410 (80.43%) | 71440 (3.29%) | 329817 (15.19%) |
| **Other Central American** | 28958 | 1260 (4.35%) | 23205 (80.13%) | 441 (1.52%) | 4052 (13.99%) |
| **Argentinean** | 268671 | 5388 (2.01%) | 240155 (89.39%) | 6253 (2.33%) | 16875 (6.28%) |
| **Bolivian** | 132343 | 1261 (0.95%) | 120534 (91.08%) | 3296 (2.49%) | 7252 (5.48%) |
| **Chilean** | 143088 | 2825 (1.97%) | 130445 (91.16%) | 2267 (1.58%) | 7551 (5.28%) |
| **Colombian** | 1091062 | 13026 (1.19%) | 974641 (89.33%) | 25434 (2.33%) | 77961 (7.15%) |
| **Ecuadorian** | 69530121524 | 5520 (0.79%) | 578321 (83.18%) | 26719 (3.84%) | 84741 (12.19%) |
| **Paraguayan** | 611629 | 901 (4.19%) | 17740 (82.42%) | 524 (2.43%) | 2359 (10.96%) |
| **Peruvian** | 60394 | 8736 (1.43%) | 559277 (91.44%) | 14320 (2.34%) | 29296 (4.79%) |
| **Uruguayan** | 358607 | 1272 (2.11%) | 52958 (87.69%) | 1167 (1.93%) | 4997 (8.27%) |
| **Venezuelan** | 23869 | 3948 (1.10%) | 325811 (90.85%) | 10747 (3.00%) | 18101 (5.05%) |
| **Other South American** | 782687 | 329 (1.38%) | 21738 (91.07%) | 353 (1.48%) | 1449 (6.07%) |
| **Spaniard** | 782687 | 20959 (2.68%) | 687177 (87.80%) | 18854 (2.41%) | 55697 (7.12%) |
| **All Other Spanish/Hispanic/Latino** | 2046539 | 112675 (5.51%) | 1545320 (75.51%) | 77734 (3.80%) | 310810 (15.19%) |
|  |  |  |  |  |  |
| **note:** |  |  |  |  |  |
| **Total Hispanic** | 17.76% |  |  |  |  |
| **Total non-Hispanic** | 82.24% |  |  |  |  |

^a^N/A refers to individuals who lived in group quarters or housing units that were vacant at the time of data collection

^b^service refers to paying a cell phone company of internet service provider

^c^No responses are regarding to access at home; may not be representative of access to public internet sources

^d^Total Spanish/Hispanic/Latino includes all related origins below (Mexican, Puerto Rican, Cuban, Dominican, Costa Rican, Guatemalan, Honduran, Nicaraguan, Panamanian, Salvadoran, Other Central American, Argentinean, Bolivian, Chilean, Colombian, Ecuadorian, Paraguayan, Peruvian, Uruguayan, Venezuelan, Other South American, Spaniard, All other Spanish/Hispanic/Latino

**Table S5b. ACS 1-Year Estimates Access to the Internet in 2019 According to the Public Use Microdata Sample by Hispanic Ethnicity**

|  |  |  |  |  |  |
| --- | --- | --- | --- | --- | --- |
| **Source: ACS 1-Year Estimates Public Use Microdata Sample 2019** | | | | | |
| Weight used: PWGTP | | | | | |
| **Access to the Internet (ACCESS)** | | | | | |
| **Recoded detailed Hispanic origin** | Total | N/A^a^ | Yes, by paying a service^b^ | Yes, without paying a service | No access^c^ |
| **-> Total** | 328239523 | 8084362 (2.46%) | 288656692 (87.94%) | 6924870 (2.11%) | 24573599 (7.49%) |
| **Not Spanish/Hispanic/Latino** | 267754746 | 6956137 (2.60%) | 236221124 (88.22%) | 5517231 (2.06%) | 19060254 (7.12%) |
| **Total Spanish/Hispanic/Latino^4^** | 60484777 | 1128225 (1.87%) | 52435568 (86.69%) | 1407639 (2.33%) | 5513345 (9.12%) |
| **Mexican** | 37185876 | 598560 (1.61%) | 32218744 (86.64%) | 851890 (2.29%) | 3516682 (9.46%) |
| **Puerto Rican** | 5846497 | 143254 (2.45%) | 5118425 (87.55%) | 138258 (2.36%) | 446560 (7.64%) |
| **Cuban** | 2381954 | 41861 (1.76%) | 2058265 (86.41%) | 68368 (2.87%) | 213460 (8.96%) |
| **Dominican** | 2085488 | 32331 (1.55%) | 1836537 (88.06%) | 54849 (2.63%) | 161771 (7.76%) |
| **Costa Rican** | 169269 | 3649 (2.16%) | 153764 (90.84%) | 3707 (2.19%) | 8149 (4.81%) |
| **Guatemalan** | 1655606 | 17506 (1.06%) | 1324030 (79.97%) | 46549 (2.81%) | 267521 (16.16%) |
| **Honduran** | 1073904 | 18338 (1.71%) | 886382 (82.54%) | 25169 (2.34%) | 144015 (13.41%) |
| **Nicaraguan** | 432579 | 4976 (1.15%) | 379299 (87.68%) | 12001 (2.77%) | 36303 (8.39%) |
| **Panamanian** | 193274 | 4156 (2.15%) | 178062 (92.13%) | 1661 (0.86%) | 9395 (4.86%) |
| **Salvadoran** | 2343993 | 26518 (1.13%) | 2041153 (87.08%) | 45662 (1.95%) | 230660 (9.84%) |
| **Other Central American** | 59512 | 646 (1.09%) | 49894 (83.84%) | 962 (1.62%) | 8010 (13.46%) |
| **Argentinean** | 308116 | 5164 (1.68%) | 291457 (94.59%) | 5278 (1.71%) | 6217 (2.02%) |
| **Bolivian** | 135191 | 348 (0.26%) | 127359 (94.21%) | 1614 (1.19%) | 5870 (4.34%) |
| **Chilean** | 158927 | 2746 (1.73%) | 152558 (95.99%) | 1680 (1.06%) | 1943 (1.22%) |
| **Colombian** | 1238369 | 15478 (1.25%) | 1157582 (93.48%) | 22005 (1.78%) | 43304 (3.50%) |
| **Ecuadorian** | 707719 | 9297 (1.31%) | 643842 (90.97%) | 12676 (1.79%) | 41904 (5.92%) |
| **Paraguayan** | 30061 | 89 (0.30%) | 29444 (97.95%) | 351 (1.17%) | 177 (0.59%) |
| **Peruvian** | 652465 | 7505 (1.15%) | 601660 (92.21%) | 12717 (1.95%) | 30583 (4.69%) |
| **Uruguayan** | 73233 | 201 (0.27%) | 70483 (96.24%) | 608 (0.83%) | 1941 (2.65%) |
| **Venezuelan** | 538536 | 4993 (0.93%) | 490070 (91.00%) | 13037 (2.42%) | 30436 (5.65%) |
| **Other South American** | 24318 | 183 (0.75%) | 21978 (90.38%) | 521 (2.14%) | 1636 (6.73%) |
| **Spaniard** | 846127 | 19133 (2.26%) | 768999 (90.88%) | 11150 (1.32%) | 46845 (5.54%) |
| **All Other Spanish/Hispanic/Latino** | 2343763 | 171293 (7.31%) | 1835581 (78.32%) | 76926 (3.28%) | 259963 (11.09%) |
|  |  |  |  |  |  |
| **note:** |  |  |  |  |  |
| **Total Hispanic** | 18.43% |  |  |  |  |
| **Total non-Hispanic** | 81.57% |  |  |  |  |

^a^N/A refers to individuals who lived in group quarters or housing units that were vacant at the time of data collection

^b^service refers to paying a cell phone company of internet service provider

^c^No responses are regarding to access at home; may not be representative of access to public internet sources

^d^Total Spanish/Hispanic/Latino includes all related origins below (Mexican, Puerto Rican, Cuban, Dominican, Costa Rican, Guatemalan, Honduran, Nicaraguan, Panamanian, Salvadoran, Other Central American, Argentinean, Bolivian, Chilean, Colombian, Ecuadorian, Paraguayan, Peruvian, Uruguayan, Venezuelan, Other South American, Spaniard, All other Spanish/Hispanic/Latino

**Table S5c. ACS 1-Year Estimates Access to the Internet in 2021 According to the Public Use Microdata Sample by Hispanic Ethnicity**

| **Source: ACS 1-Year Estimates Public Use Microdata Sample 2021** | | | | | |
| --- | --- | --- | --- | --- | --- |
| Weight used: PWGTP | | | | | |
| **Access to the Internet (ACCESSINET)** | | | | | |
| **Recoded detailed Hispanic origin** | Total | N/A^a^ | Yes, by paying a service^b^ | Yes, without paying a service | No access^c^ |
| **Total** | 331893745 | 7760859 (2.34%) | 301737502 (90.91%) | 6328745 (1.91%) | 16066639 (4.84%) |
| **Not Spanish/Hispanic/Latino** | 269363212 | 6755143 (2.51%) | 244734198 (90.86%) | 5004337 (1.86%) | 12869534 (4.78%) |
| **Total Spanish/Hispanic/Latino^d^** | 62530533 | 1005716 (1.61%) | 57003304 (91.16%) | 1324408 (2.12%) | 3197105 (5.11%) |
| **Mexican** | 37249675 | 520237 (1.40%) | 34070010 (91.46%) | 783778 (2.10%) | 1875650 (5.04%) |
| **Puerto Rican** | 5801064 | 105552 (1.82%) | 5286988 (91.14%) | 131245 (2.26%) | 277279 (4.78%) |
| **Cuban** | 2402116 | 38332 (1.60%) | 2166202 (90.18%) | 58667 (2.44%) | 138915 (5.78%) |
| **Dominican** | 2421393 | 27246 (1.13%) | 2235808 (92.34%) | 52818 (2.18%) | 105521 (4.36%) |
| **Costa Rican** | 183070 | 2555 (1.40%) | 174907 (95.54%) | 2532 (1.38%) | 3076 (1.68%) |
| **Guatemalan** | 1770604 | 18814 (1.06%) | 1511965 (85.39%) | 40778 (2.30%) | 199047 (11.24%) |
| **Honduran** | 1123243 | 11711 (1.04%) | 977720 (87.05%) | 27011 (2.40%) | 106801 (9.51%) |
| **Nicaraguan** | 452590 | 4046 (0.89%) | 416153 (91.95%) | 11921 (2.63%) | 20470 (4.52%) |
| **Panamanian** | 235136 | 4450 (1.89%) | 215096 (91.48%) | 7403 (3.15%) | 8187 (3.48%) |
| **Salvadoran** | 2486513 | 17928 (0.72%) | 2301504 (92.56%) | 45154 (1.82%) | 121927 (4.90%) |
| **Other Central American** | 27044 | 665 (2.46%) | 25329 (93.66%) | 21 (0.08%) | 1029 (3.80%) |
| **Argentinean** | 290013 | 3524 (1.22%) | 271612 (93.66%) | 7778 (2.68%) | 7099 (2.45%) |
| **Bolivian** | 127381 | 576 (0.45%) | 122686 (96.31%) | 1565 (1.23%) | 2554 (2.01%) |
| **Chilean** | 191055 | 3352 (1.75%) | 179861 (94.14%) | 3518 (1.84%) | 4324 (2.26%) |
| **Colombian** | 1422553 | 17776 (1.25%) | 1342697 (94.39%) | 29947 (2.11%) | 32133 (2.26%) |
| **Ecuadorian** | 831552 | 7443 (0.90%) | 782190 (94.06%) | 10683 (1.28%) | 31236 (3.76%) |
| **Paraguayan** | 29244 | 745 (2.55%) | 27958 (95.60%) | 265 (0.91%) | 276 (0.94%) |
| **Peruvian** | 707922 | 7124 (1.01%) | 667862 (94.34%) | 13247 (1.87%) | 19689 (2.78%) |
| **Uruguayan** | 63877 | 1425 (2.23%) | 58747 (91.97%) | 1748 (2.74%) | 1957 (3.06%) |
| **Venezuelan** | 643470 | 4542 (0.71%) | 610146 (94.82%) | 9250 (1.44%) | 19532 (3.04%) |
| **Other South American** | 40927 | 232 (0.57%) | 39126 (95.60%) | 535 (1.31%) | 1034 (2.53%) |
| **Spaniard** | 992848 | 17120 (1.72%) | 931401 (93.81%) | 16244 (1.64%) | 28083 (2.83%) |
| **All Other Spanish/Hispanic/Latino** | 3037243 | 190321 (6.27%) | 2587336 (85.19%) | 68300 (2.25%) | 191286 (6.30%) |
|  |  |  |  |  |  |
| **note:** |  |  |  |  |  |
| **% total Hispanic** | 18.84% |  |  |  |  |
| **% total non-Hispanic** | 81.16% |  |  |  |  |

^a^N/A refers to individuals who lived in group quarters or housing units that were vacant at the time of data collection

^b^service refers to paying a cell phone company of internet service provider

^c^No responses are regarding to access at home; may not be representative of access to public internet sources

^d^Total Spanish/Hispanic/Latino includes all related origins below (Mexican, Puerto Rican, Cuban, Dominican, Costa Rican, Guatemalan, Honduran, Nicaraguan, Panamanian, Salvadoran, Other Central American, Argentinean, Bolivian, Chilean, Colombian, Ecuadorian, Paraguayan, Peruvian, Uruguayan, Venezuelan, Other South American, Spaniard, All other Spanish/Hispanic/Latino

**Figure S1. Clinical Sites of The DCM Consortium for the DCM Precision Medicine Study.**

Shown are the 25 clinical sites of the DCM Consortium who enrolled probands and family members for the DCM Precision Medicine Study. The Ohio State University site served as an enrolling site as well as the coordinating center.

**Figure S2. Beta testing phases of The DCM Project Portal**

**
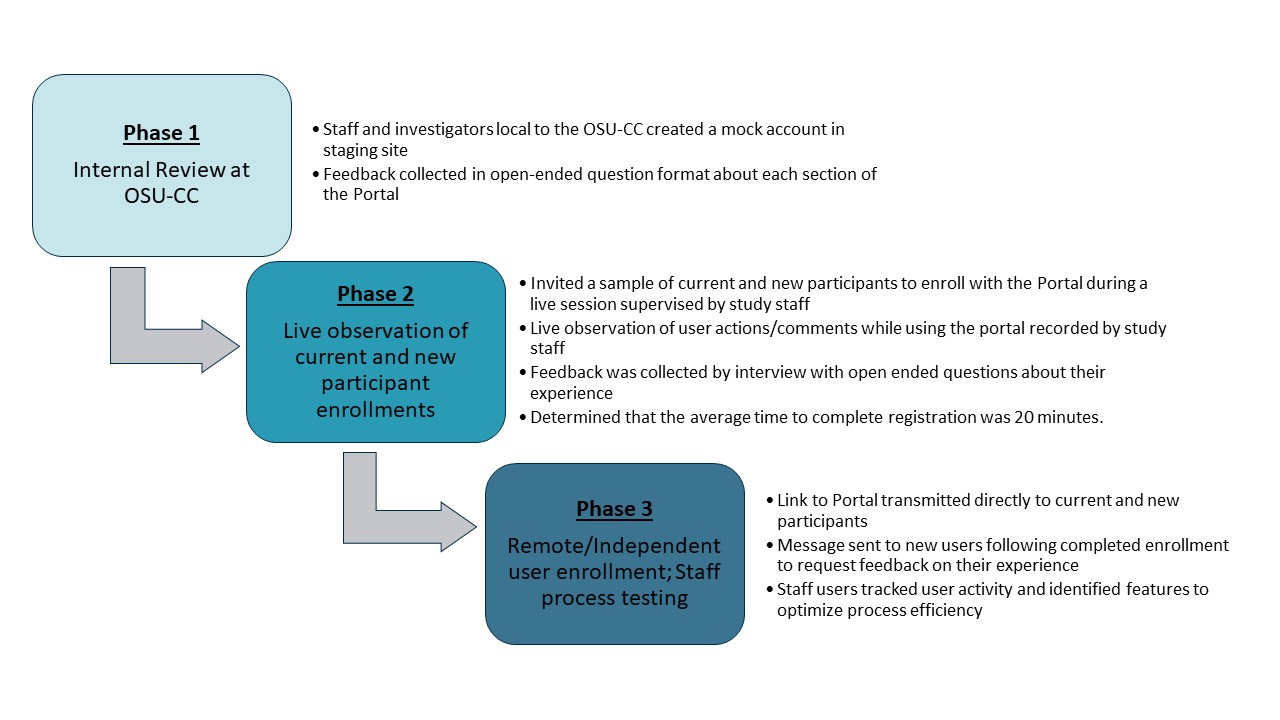
**

**Figure S2.** Three phases of beta testing were conducted before opening the DCM Project Portal for active service. Phase 1 included an internal review of staff and investigators familiar with the research project. Staff and investigators created mock accounts and offered feedback in an open-ended question form. After incorporating feedback, Phase 2 included a sample of participant users, including those previously consented to the study as well as newly invited. Individuals were observed going through the portal process live while a staff took notes of the actions and comments made during the experience. Feedback was collected directly once the process was completed by structured interview by the observing study staff member. Adjustments were made according to Phase 2 results before beginning Phase 3, where select participants (new and previously consented) were invited to create an account but this time independently. Progress was tracked by staff and feedback collected through the messaging system upon completion. Phase 3 findings included requests from both users and staff to maximize usability and efficiency.
